# From the Andes to the desert: 16S rRNA metabarcoding characterization of aquatic bacterial communities in the Rimac river, the main source of water for Lima, Peru

**DOI:** 10.1371/journal.pone.0250401

**Published:** 2021-04-22

**Authors:** Pedro E. Romero, Erika Calla-Quispe, Camila Castillo-Vilcahuaman, Mateo Yokoo, Hammerly Lino Fuentes-Rivera, Jorge L. Ramirez, André Ampuero, Alfredo J. Ibáñez, Paolo Wong

**Affiliations:** 1 Departamento de Ciencias Biológicas y Fisiológicas, Facultad de Ciencias y Filosofia, Universidad Peruana Cayetano Heredia, Lima, Peru; 2 Instituto de Ciencias Ómicas y Biotecnología Aplicada (ICOBA), Pontificia Universidad Católica del Peru, Lima, Peru; 3 Departamento de Ciencias de la Medicina, Facultad de Medicina Humana, Universidad de Piura, Lima, Peru; 4 Departamento de Biología Celular y Genética, Facultad de Ciencias Biológicas, Universidad Nacional Mayor de San Marcos, Lima, Peru; 5 Departamento de Malacología y Carcinología, Museo de Historia Natural, Universidad Nacional Mayor de San Marcos, Lima, Peru; Universidade Estadual de Goias - Campus de Ciencias Exatas e Tecnologicas Henrique Santillo, BRAZIL

## Abstract

The Rimac river is the main source of water for Lima, Peru’s capital megacity. The river is constantly affected by different types of contamination including mine tailings in the Andes and urban sewage in the metropolitan area. In this work, we aim to produce the first characterization of aquatic bacterial communities in the Rimac river using a 16S rRNA metabarcoding approach which would be useful to identify bacterial diversity and potential understudied pathogens. We report a lower diversity in bacterial communities from the Lower Rimac (Metropolitan zone) in comparison to other sub-basins. Samples were generally grouped according to their geographical location. Bacterial classes Alphaproteobacteria, Bacteroidia, Campylobacteria, Fusobacteriia, and Gammaproteobacteria were the most frequent along the river. *Arcobacter cryaerophilus* (Campylobacteria) was the most frequent species in the Lower Rimac while *Flavobacterium succinicans* (Bacteroidia) and *Hypnocyclicus* (Fusobacteriia) were the most predominant in the Upper Rimac. Predicted metabolic functions in the microbiota include bacterial motility and quorum sensing. Additional metabolomic analyses showed the presence of some insecticides and herbicides in the Parac-Upper Rimac and Santa Eulalia-Parac sub-basins. The dominance in the Metropolitan area of *Arcobacter cryaerophilus*, an emergent pathogen associated with fecal contamination and antibiotic multiresistance, that is not usually reported in traditional microbiological quality assessments, highlights the necessity to apply next-generation sequencing tools to improve pathogen surveillance. We believe that our study will encourage the integration of omics sciences in Peru and its application on current environmental and public health issues.

## Introduction

Worldwide, water quality problems are associated with poverty conditions and lack of efficient sanitation, especially in developing countries [1]. These problems are recurrent in highly populated cities where most of the waste is directly washed to nearby rivers [2]. Lima, the capital city of Peru, is the second largest desert city in the world [3]. Its Metropolitan area is inhabited by more than 10 million people creating enormous challenges for environmental and public health [4]. For instance, it has been shown that water quality in Lima is a significant risk factor for pathogenic infections in children [5].

The Rimac river is the main source of drinking water for the Lima Metropolitan area. Recently, more than 700 pollution sources were identified by the Peruvian National Authority of Water (ANA) [6]. The river is constantly polluted by mine tailings in the Upper Rimac closer to the central Peruvian Andes, by agricultural wastewater in its middle region, and by industrial wastewater and urban sewage in the Lower Rimac within the Metropolitan area nearby the Pacific Ocean [7]. The lack of an efficient wastewater treatment in the Metropolitan region promotes the presence of potentially pathogenic bacteria such as *Escherichia coli* or *Salmonella typhi*, associated with fever and diarrhea symptoms [2, 8].

Traditionally, assessments of water quality and bacterial contamination in Peru are focused on evaluating the presence of common coliforms (e. g. *Citrobacter*, *Enterobacter*, *Escherichia*, *Klebsiella*) [9]. However, there are a plethora of likely pathogens that are not currently studied using classic methods because of taxonomic assignation problems or the existence of non-culturable phenotypes [10]. In recent years, advances in the study of bacterial communities and diversity have occurred because of the improvement of next-generation sequencing (NGS) methodologies. One of these techniques, 16S rRNA metabarcoding, uses a fragment of the 16S ribosomal gene to obtain a diversity profile of the bacterial community in a specific environment. Therefore, it has been used to study different types of samples from the internal microbiota of several species, including humans to environmental community surveys [11].

16S rRNA metabarcoding has been also used to study bacterial communities from several rivers. For instance, a study in the Danube river, which crosses many countries from central Europe, found a higher microbial community richness in the Upper basin, while in the Lower basin, there was a predominance of only a few free-living and particle associated bacteria [12]. Studies in rivers have also looked for the occurrence of potential pathogens. A report from the river Tama (Tokyo, Japan) showed that the predominant bacteria genus was Flavobacterium (Bacteroidia), a freshwater fish pathogen [13]. Recently, a study in the Pinheiros river (Sao Paulo, Brazil), one of the most polluted Brazilian rivers, found the predominance of *Arcobacter cryaerophylus* (Campylobacteria). This species is considered an emerging pathogen and an indicator of fecal contamination [14, 15].

Information from metabolomics provides additional insight into chemical contaminants in water that may influence bacterial diversity and would be useful to indicate the health status of an aquatic ecosystem and understand interactions between microbial communities and their environment [16, 17]. For instance, a study in the Brisbane river (Australia) found that human interference was associated with a population increase of Actinomycetes and Pseudomonadales. This increment was linked to higher levels of sugar alcohols, short-chain fatty acids and aromatic amino acids which contribute towards biofilm production [16].

Aquatic bacteria play a key role in water ecosystems being involved in biogeochemical processes and attenuation of biological and chemical pollutants [18]. Although some bacteria can have an adverse impact causing water quality deterioration and public health problems. We agree with Paruch et al. [18] that the understanding of aquatic microbial diversity, composition, and dynamics is important for water quality assessments and management strategies for sustainable ecosystem functions.

Thus, we aim to provide for the first time an overview of the microbiota from the Rimac river. We aim to compare diversity patterns from the Rimac river sub-basins and show differences in the bacteria composition from Andean to Metropolitan areas. Our study is exploratory and could be used as evidence for future rapid biodiversity surveys or water quality assessments focused on microbial diversity.

## Materials & methods

### Area of study and sampling

**Fig 1:**
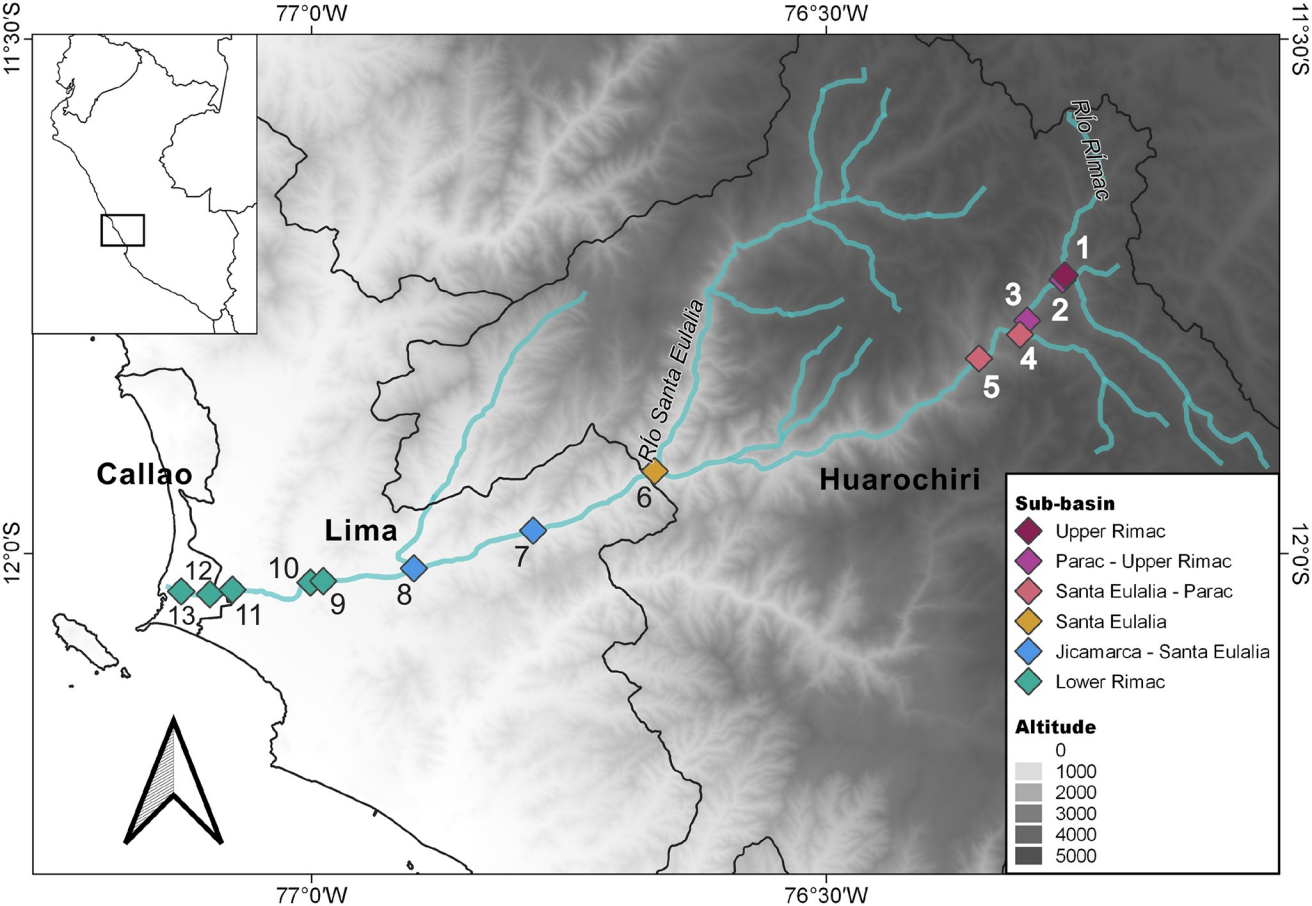
Area of study and sampling localities. The Rimac basin is situated between the central Peruvian Andes and the Pacific Ocean. The river runs across Callao, Huarochiri and Lima provinces in the Lima region. We sampled in different localities from the following sub-basins: Upper Rimac, 1. Chicla; Parac-Upper Rimac, 2. San Mateo, 3. Tamboraque; Santa Eulalia-Parac, 4. Huanchor, 5. Chacahuaro; Santa Eulalia, 6. Santa Eulalia; Jicamarca-Santa Eulalia, 7. Chaclacayo, 8. Huachipa; Lower Rimac (Metropolitan area), 9. Libertadores, 10. Nuevo, 11. Universitaria, 12. Faucett, 13. Gambetta.

In January 2020, we sampled river water in 13 points along the extension of the Rimac river, within provinces of Huarochiri, Lima and Callao (Fig 1). According to the Peruvian National Authority of Water, the Rimac basin is divided in nine sub-bains [19]. We sampled from six sub-basins, namely, Upper Rimac (Chicla), Parac-Upper Rimac (San Mateo, Tamboraque), Santa Eulalia-Parac (Huanchor, Chacahuaro), Santa Eulalia (Santa Eulalia), Jicamarca-Santa Eulalia (Huachipa, Chaclacayo), Lower Rimac, near main city bridges (Libertadores, Nuevo, Universitaria, Faucett and Gambetta). Sterile plastic bottles were used to sample approximately 1.5 L of superficial water. When we sampled from a bridge, we attached the plastic bottle to another flask linked to a 30 m rope. In this case, we sampled superficial water (ca. 50 cm below the surface) from the middle of the bridge. Water samples were transported to the lab in coolers and were processed in the same day. Ten milliliters of water were removed for the metabolomics study. The rest of the sample was filtered river using 0.2 μM Sterivex filters for DNA extraction.

#### DNA isolation, amplification, and preparation of genomic libraries

Total DNA was isolated from the Sterivex filter using the PowerWater DNA kit (Qiagen) following manufacturer’s instructions. Additionally, DNA isolation was performed from two empty Sterivex filters, both samples were used as blanks. Then, we quantified DNA quality using a Qubit 3.0 (Thermo Fisher Scientific, USA) fluorometer. We followed the standard Illumina 16S rRNA genomic library preparation protocol [20] which consisted in the amplification of the 16S rRNA V3-V4 region (ca. 420 bp). A second amplification was performed to attach oligo adapters (indexes) to each amplicon sample. Indexes were informative to differentiate samples after the sequencing step. Each step was followed by amplicon cleaning using AMPure XP beads (Beckman Coulter, USA). In the final step, all samples were pooled and sequenced using the MiSeq v3 Reagent Kit (Illumina, USA) on the MiSeq instrument (Illumina). Raw sequences from the genomic libraries were deposited in the NCBI BioProject database (accession number: PRJNA646070) (S1 Table).

### Bioinformatics and data analyses

Sequencing reads per each sample were analyzed using DADA2 [21] in R v3.6.3 [22]. Reads from replicate samples, with the exception of Chacahuaro and Universitaria localities were also integrated in the analysis. We filtered reads using the parameter maxEE = 2 (maximum number of expected errors) for quality threshold as suggested before [23, 24]. DADA2 was used to infer Amplicon Sequence Variants (ASV) which are groups of identical sequences. We also used DADA2 for taxonomic assignment, comparing ASV sequences against the ribosomal database SILVA v.138 [25] with the function “*assignTaxonomy*”. Identification up to the species (or multispecies) was done using the function “*assignSpecies*”. Only two ASVs were identified in the blanks corresponding to ASV_2999 (*Wolbachia*) and ASV_3029 (*Rickettsia*) and were removed from all samples in further analyses. The code for bioinformatic analyses can be accessed from GitHub [26]. We used the Chao1 index, a non-parametric richness index that calculates the minimal number of taxa present in a sample, and the Shannon index, which estimates the taxa diversity in a sample taking into account both abundance and evenness [27]. Both indexes were estimated using the package phyloseq [28].

To compare Chao1 and Shannon indices from each sub-basin we used the one-way ANOVA and the Tukey’s post hoc tests. Assumptions of normality and heteroscedasticity were checked with Shapiro-Wilks and Breusch-Pagan tests, implemented in the packages agricolae [29] and car [30], respectively [31–33]. To compare beta diversity among localities, we normalized the ASV matrix using the variance stabilizing transformation (VST) method available in the package DESeq2 [34], as suggested before for microbiome analyses [35]. Then, we used a multidimensional scaling (MDS) approach based on using a Bray-Curtis similarity matrix to compare beta diversity among localities.

In addition, we used the taxonomic and occurrence information to produce a stacked bar plot of the most predominant classes. Then, we explored the most predominant genera using the package ampvis2 [36]. Next, we selected ASV with more than 1 000 counts and a taxonomic assignment until species level. We used this information to look for potential human and other animal pathogens in the Risk Group database [37], specifically if predicted species are pathogens for humans or other animals. Finally, we used Piphillin [38, 39] to predict functional content based on the frequency of the 16S rRNA sequences comparing them to annotated genomes in the Kyoto Encyclopedia of Genes and Genomes (KEGG) database. Predicted KEGG orthologues (KO) occurrence was retrieved from KEGG (May 2020) using a 97% cutoff threshold to create a gene feature table. We used the 10 most frequent unique KO per locality, then we grouped localities by sub-basin and created a Venn diagram to look for shared KOs for each region using DeepVenn [40].

Finally, we did a redundance analysis (RDA) using information of environmental parameters (pH, temperature, electric conductivity [EC], nitrate concentration) obtained from a report of the Peruvian National Authority of Water (ANA) [41] (S2 Table). Eight sample sites were located to the near points evaluated in the ANA report so environmental information could be comparable. Significant identified compounds from the metabolomic analysis were also added to the RDA. The RDA was performed using a Bray-Curtis distance matrix estimated from logarithmic transformed count data that prevents placing too much weight on extremely high values of ASVs. Forward selection was carried out to calculate the relative significances of abiotic variables, using the package adespatial [42] in R. We also performed a Spearman correlation in R among environmental parameters, significant identified compounds in the metabolomic analyses, and bacterial classes frequency in the sampling locations. P-values were corrected for multiple comparisons using the FDR correction.

### Metabolomic analyses

Ten milliliters of each water sample and Milli Q water (Blank) were filtered through a Nalgene^™^ 0.22 μm syringe filter (Thermo Scientific) to remove suspended particulates. Each water sample was immediately extracted with dichloromethane (ACS grade, J.T. Baker) (3 times x 10 mL). After that, dichloromethane extracts were concentrated in vacuo and placed in a vial with 150 μL of dichloromethane and 2 μL of 26.7 ppb of toluene (GC grade, Sigma-Aldrich) (internal standard). The standard solution consisted in 2 ppm of toluene in dichloromethane, 2 μL of this solution was diluted with 150 μL dichloromethane. All water samples were analyzed except Santa Eulalia (6), Chaclacayo (7), Huachipa (8) and Gambetta (13) because these water samples were used completely in the previous filtering step. Water samples, blanks and internal standards were analyzed by gas chromatography (GC) coupled to an APPI-Q-Exactive HF mass spectrometer (Thermo Fisher Scientific, USA). GC was equipped with a DB-5 column (30 m x 0.25 mm i.d., 0.25 μm thickness film). The oven temperature was programmed as follows: aliquots of 2 μL sample were injected at 45 °C for 2 min, increase at 10 °C/min until 270 °C, and hold for 7.5 min. Injections were made in splitless-mode with helium as the carrier gas (1.5 mL/min), injector temperature at 270 °C, and detector temperature at 270 °C. High-accuracy MS data were acquired in positive data-dependent acquisition (DDA) with scan range m/z 50–750. Raw data from this GC-MS experiment was first converted into ABF format. Then, peak spotting was performed by exploring retention time and accurate mass. MS-DIAL v4.16 provided peak alignments of all samples and normalizes data based on TIC (total ion current). Final filtering was performed with a principal component analysis (PCA, p-anova < 0.05) using MATLAB vR2019b to track data quality, reduce the data dimensionality, identify potential outliers in the dataset, as well as to identify sample clusters. Additionally, the software Compound Discovery was used for tentative identification using MS/MS data and comparing our results against several databases such as ChemBioFinder, Chemspider, Kegg, LipidBank, LipidMaps, Metlin and NIST. Besides, a PCA was performed to observe differences in mass profiles among samples group.

### Ethics statement

The study did not involve experiments in humans nor animal subjects. We did not sample in protected areas nor protected species were sampled.

## Results

### Characterization of bacterial communities in the Rimac sub-basins

**Fig 2:**
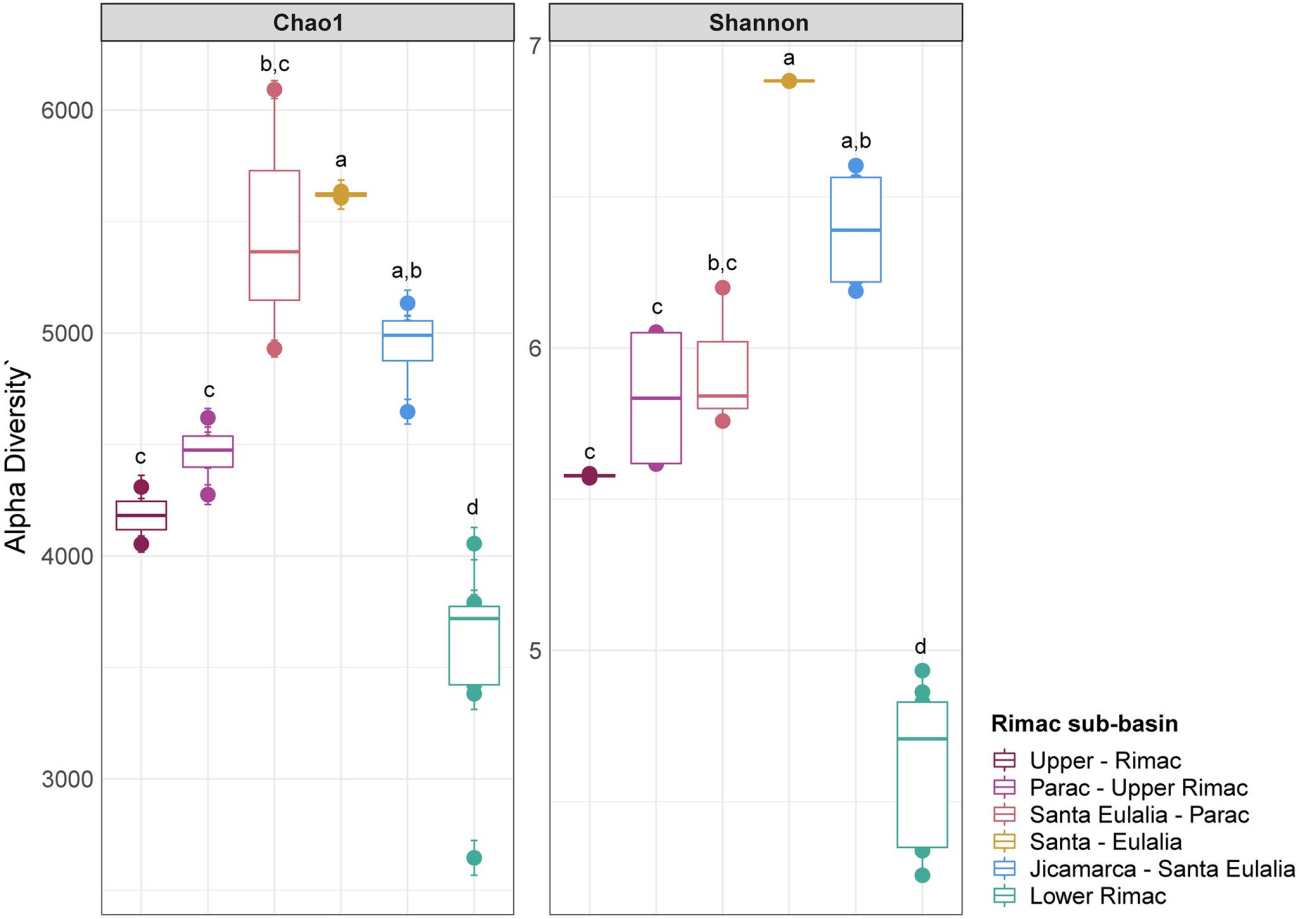
Alpha diversity indexes, Chao1 (richness) and Shannon (diversity) in the samples. The Lower Rimac localities consistently showed lower richness and diversity values in comparison to other sub-basins. Letters above boxplots represent significant differences (p<0.05) between average values of each sub-basin.

**Fig 3:**
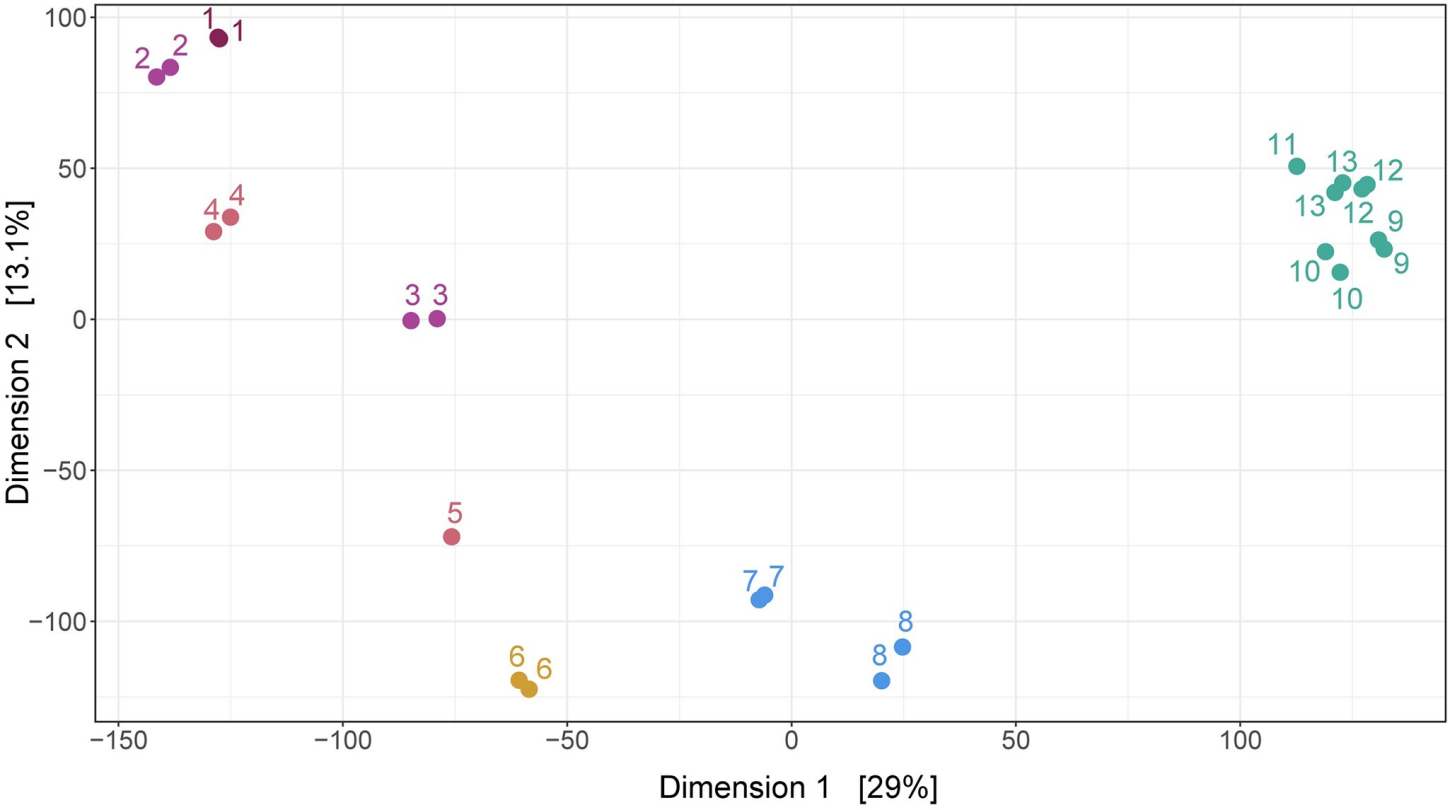
Multi-Dimensional Scaling (MDS). Principal coordinate analysis (PCoA) of ASVs shows similarities among groups of samples. Lower Rimac localities (green) clustered together and are less similar to other sub-basins. Samples are numbered according to the information in Fig 1.

16S rRNA metabarcoding produced an average of 244 393 [105 680–287437] reads per sample. After discarding low quality sequences and merging forward and reverse reads, we obtained an average of 92 064 [39 496–124 58] final reads per sample (S1 Table). From this subset, we identified a total of 23 682 ASV. The first 268 ASV were represented by more than 1 000 reads and the first 19, by more than 10 000 reads (S3 Table). Richness (Chao1 index) and diversity (Shannon index) was significantly lower for the Lower Rimac sub-basin in comparison to the other sub-basins (Fig 2). In the MDS analysis, samples from the Lower Rimac clustered tightly together and are less similar to other sub-basins. Samples from Parac-Upper Rimac and Santa Eulalia-Parac sub-basins did not clustered together. Aquatic bacteria community diversity seems higher in the Upper and middle Rimac than in the Lower Rimac (Fig 3).

**Fig 4:**
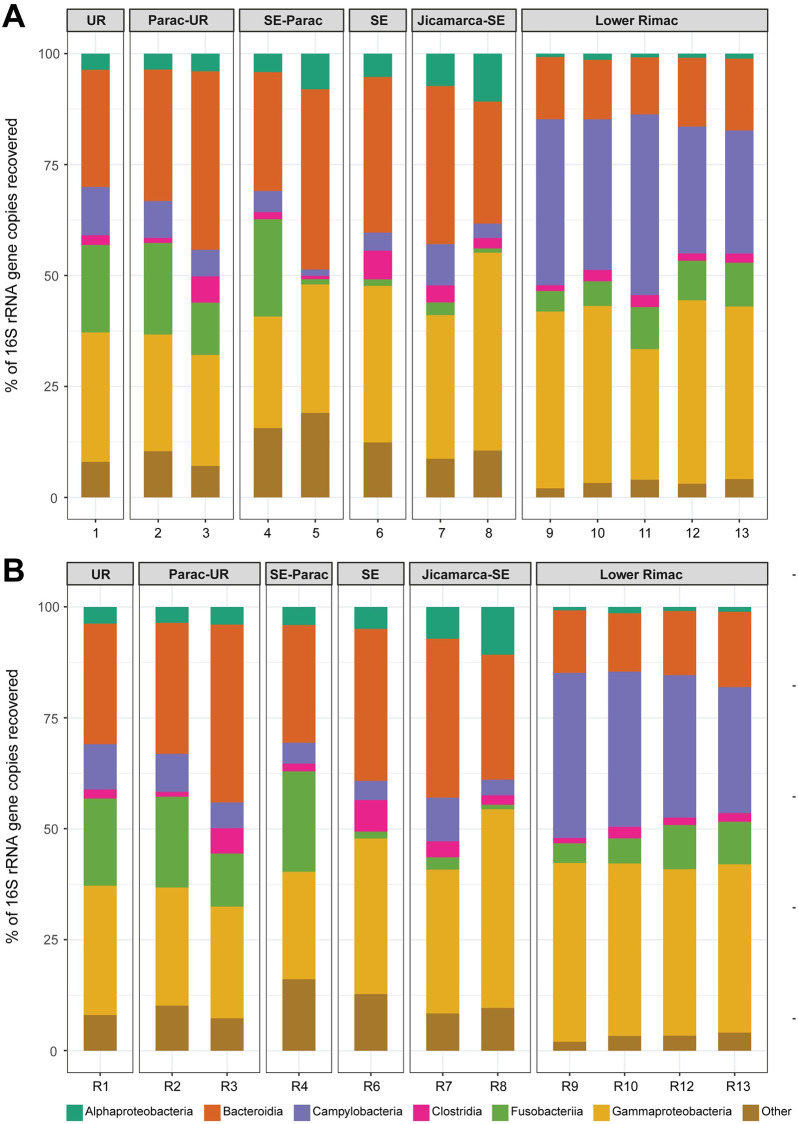
Stack barplot of the frequencies from the most common bacterial classes in each locality. Samples are numbered according to the information in Fig 1. UR: Upper Rimac, SE: Santa Eulalia. A. Samples, B. Replicates.

**Fig 5:**
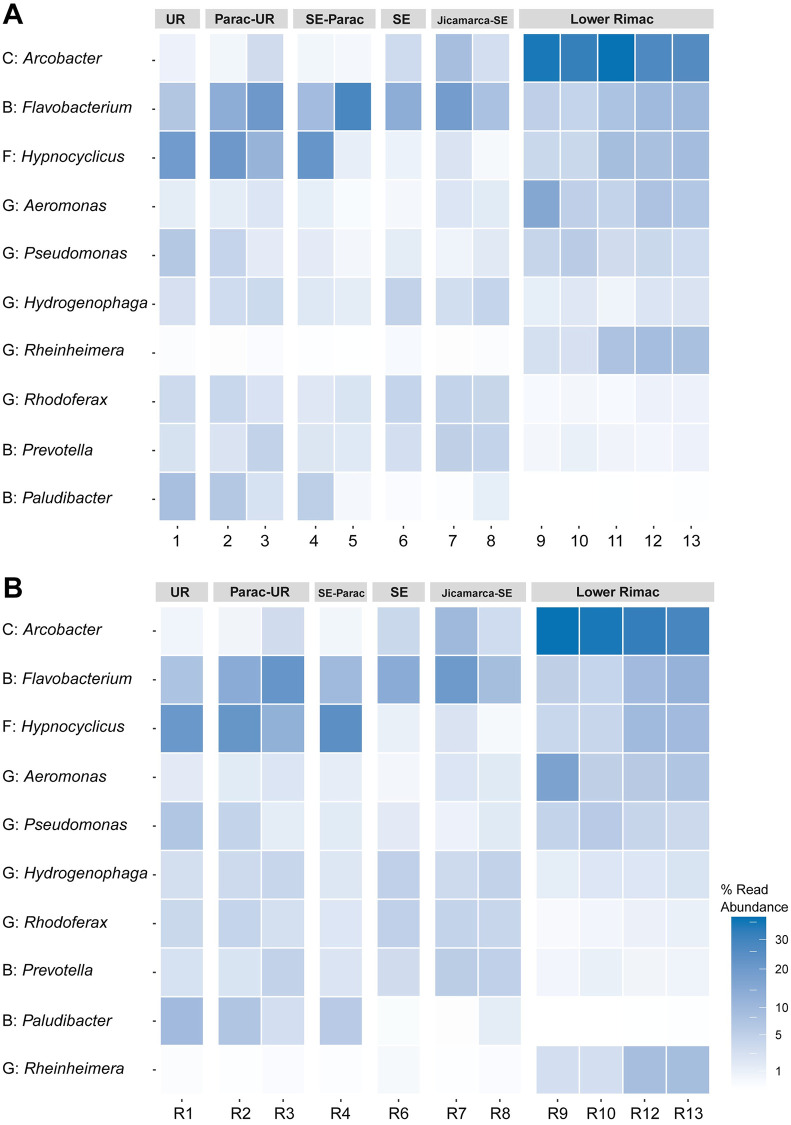
Heatmap of the abundance from the ten most frequent bacterial genera. Classes codes: B, Bacterioidota; C, Campylobacteria; F, Fusobacteriia; G, Gammaproteobacteria. Samples are numbered according to the information in Fig 1. UR: Upper Rimac, SE: Santa Eulalia. A. Samples, B. Replicates.

Phyla Bacteroidota, Campylobacterota, Firmicutes, Fusobacteriota and Proteobacteria represented 75.75% (18 078 from 23 864) of the total ASVs (S4 Table). Taxonomic identification of ASV was higher for phylum, class, order, and family ranks (95–99%), and moderate for genus and species ranks (84 and 66%, respectively) (S4 Table). Classes Alphaproteobacteria, Bacteroidia, Campylobacteria, Clostridia, Fusobacteriia, and Gammaproteobacteria were the consistently common along all zones (Fig 4). Fusobacteriia declines from Chacahuaro (Santa Eulalia-Parac) to Huachipa (Jicamarca- Santa Eulalia). Campylobacteria frequency rises in the Lower Rimac zone (Metropolitan area). The most frequent genera (Fig 5) in the Upper Rimac, Parac-Upper Rimac, and Santa Eulalia-Parac sub-basins were *Hypnocyclicus* (ASV2) (Fusobacteriia) and Flavobacterium (ASV3) (Bacteroidia), the latter was also predominant in Santa Eulalalia and Jicamarca-Santa Eulalia sub-basins. In the Lower Rimac, we found a dominance of *Arcobacter* (Campylobacteria). Human pathogenic bacteria identified to species level were *Arcobacter cryaerophylus* and *Prevotella copri* (S5 Table), and to the genus level were *Aeromonas*, *Escherichia*, *Pseudomonas* and *Shigella*. Other animal pathogens identified were *Flavobacterium succinicans* and *Faecalibacterium prausnitzii*.

Piphillin functional prediction of the top unique KEGG orthologies (KO) for each locality revealed 7 KO in the Upper Rimac sub-basin, 11 in Parac-Upper Rimac, 18 in Santa Eulalia-Parac, 10 in Santa Eulalia, 12 in Jicamarca-Santa Eulalia, and 9 in the Lower Rimac (S6 Table). The Venn diagram shows two KO (K02030, K03406) shared by the six sub-basin zones, and two KO (K05874, K01999) were shared among all sub-basins except the Lower Rimac (S1 Fig). The most abundant predicted pathways shared by the six sub-basins (S6 Table) were related to the ATP-binding cassette transporters (polar amino acid transport system substrate-binding protein, K02030), bacterial chemotaxis (methyl-accepting chemotaxis protein/MCP, K03406), and quorum sensing (K01999).

**Fig 6:**
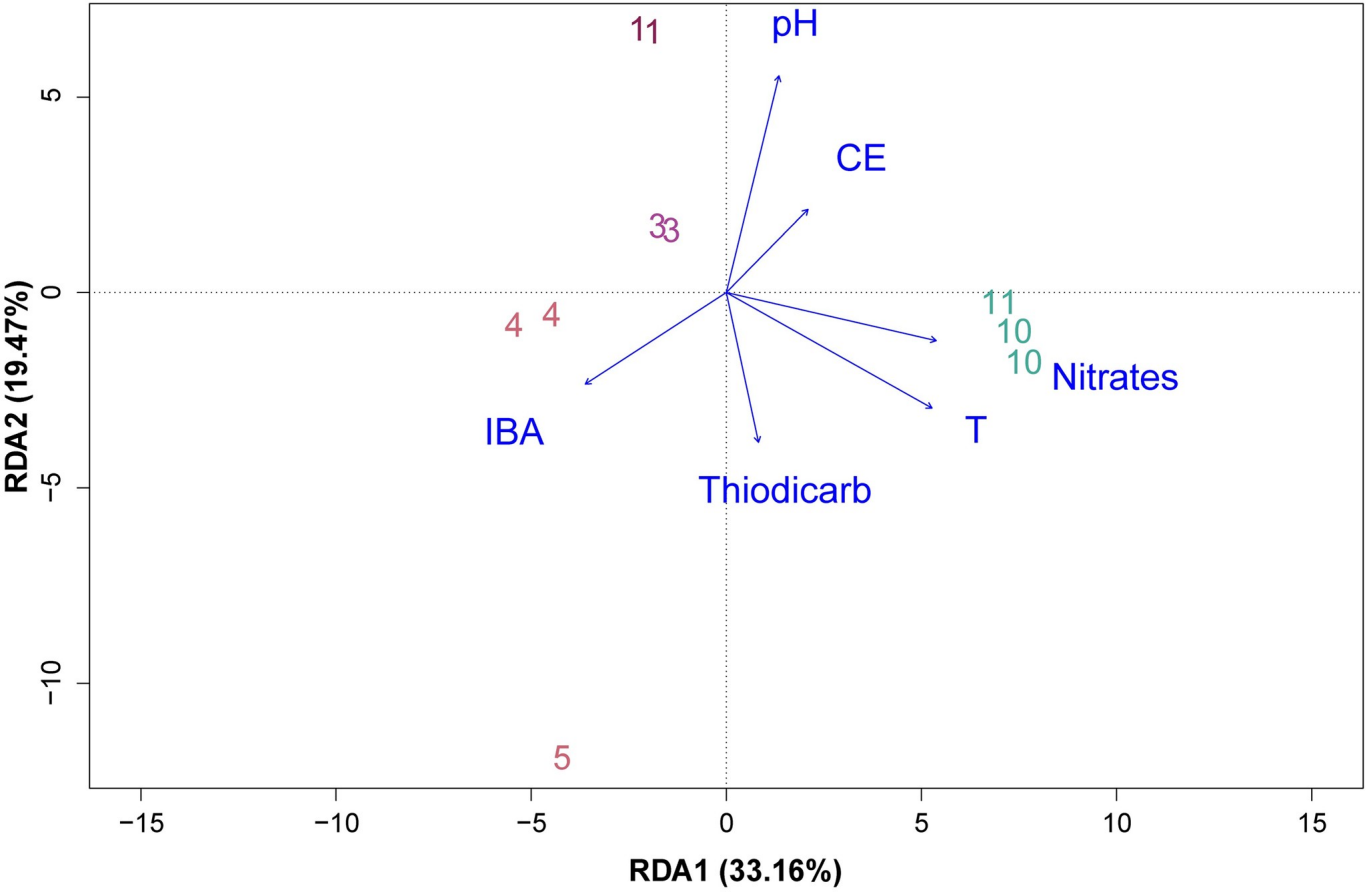
Redundance analysis (RDA) between species with environmental factors. Samples are numbered according to the information in Fig 1. Environmental data was taken from Ref. 34. T: Temperature, EC: Electrical conductivity. IBA: 3-indolebutyric acid.

The redundant analysis (RDA) showed that the first and second axes accounted for 33.16% and 19.47% respectively, of the total variation in the community, the first axis showed that microbial communities were associated with temperature and nitrates (longest arrows), while the second axis was associated to pH (Fig 6). Temperature was found to be the most significant among explanatory variables (p = 0.001). Finally, the Spearman correlation coefficient (S2 Fig) showed that Gammaproteobacteria and Campylobacteria frequencies were positively correlated with higher nitrate concentration. Gammaproteobacteria frequency seems to be positively correlated with temperature.

### Metabolomic analyses

The untargeted GC-MS (APPI) analysis in positive mode resulted in a curated data matrix, comprised of 166 features (i.e. *m/z* values) (S7 Table). Moreover, PCA performed from these results (S3 Fig), showed distinguishable separation between the Upper Rimac, Parac-Upper Rimac, Santa Eulalia-Parac, Lower Rimac, blanks and internal standard samples. Indeed, some localities from the Parac-Upper Rimac sub-basin (San Mateo, Tamboraque) and Santa Eulalia-Parac (Huanchor, Chacahuaro) were well differentiated from the other areas. However, the Upper Rimac sample (Chicla) was clustered within the Lower Rimac because of similar chemical composition. Additionally, we tentatively identified five compounds from thirteen features statistically significant (p-anova < 0.05, total score > 95%) using Compound Discovery and MS-DIAL softwares (S8 Table), such as thiodicarb (peak 93), 3-indolebutyric acid (IBA) (peak 104) and benzenemethanamine derivatives (peaks 71, 112 and 119). The loading plot of PCA coefficients from metabolomics analysis (S4 Fig) showed that peak 104 was identified in Santa Eulalia-Parac localities and peaks 71, 92, 93, 112 and 119 were identified in Parac-Upper Rimac and in Santa Eulalia-Parac localities. Whereas the Lower Rimac samples were influenced by peak 43. Additionally, peaks 22, 23, 29–31 and 87 were identified in all zones (S8 Table).

## Discussion

Our study provides the first overview of the bacterial community diversity in the Rimac river (Lima, Peru) using a 16S rRNA metabarcoding approach. We described bacterial community composition shifts along an altitude gradient (0–4 500 msl), and compared these results with environmental and metabolomics data available. We found a clear separation among bacterial communities from the Upper Rimac (> 3 000 msl) and Lower Rimac sub-basins (< 300 msl), while this was more subtle for the intermediate sub-basins. We mainly found the occurrence of phyla Bacteroidota, Campylobacterota, Fusobacteriota and Proteobacteria along the Rimac river basin. This result is similar to a previous report in the polluted Pinheiros river in Sao Paulo, Brazil [14]. The authors associated the high presence of these phyla with freshwater environments and domestic sewage sludges.

Bacterial communities in high-altitude aquatic environments are still not thoroughly studied. We found only few characterizations using 16S rRNA sequencing, especially in lakes. For instance, community profiling of Tibetan and Pyrenean lakes showed dominance of classes Actinobacteria, Alphaproteobacteria, Betaproteobacteria, and phyla Bacteroidota [43–45]. Our samples did have a major presence of the Bacteroidia class, part of the latter phylum, and class Alphaproteobacteria. However, we found no Betaproteobacteria. In the Upper Rimac and Parac-Upper Rimac sub-basins, we found a predominance of *Hypnocyclicus* and *Flavobacterium*, the latter has been related to several issues in fish such as “cold water” and *columnaris* disease [46]. In particular, *Flavobacterium succinicans*, the most frequent *Flavobacterium* (S4 Table), has been associated with bacterial gill disease in trout [47]. Additional surveys in high-altitude rivers are necessary to have a broader view of bacterial communities in these kinds of habitats.

The Lower Rimac is the essential water source for the Lima Metropolitan area, a mega-city situated on the hyper-arid Peruvian desert [4, 48]. Community composition in the Lower Rimac is influenced by similar pollution factors such as human feces and domestic sewage that are produced in the Metropolitan area [49]. This could explain why samples from the Lower Rimac looked more similar among each other than samples from other sub-basins (MDS, Fig 3). The rise of Campylobacteria frequency in the Lower Rimac is correlated with a higher nitrate concentration (S2 Table). Source of the latter are fertilizers, sewage sludge and discharges from municipal wastewater treatment [50, 51]. Thus, urban pollution in the last part of the Rimac river is influencing the bacterial diversity and the occurrence of potential pathogens.

*Arcobacter cryaerophilus*, the predominant species in the Lower Rimac, has been reported as very frequent in other rivers such as the Yangtze (China) [49], many rivers in Nepal [52], the Llobregat (Spain) [53], and the Pinheiros river [14]. As mentioned before, *Arcobacter* is an emergent enteropathogenic bacterium and an indicator of fecal contamination. It can also occur in water treatment and sewage systems [54] and survive adverse conditions imposed by food processing and storage [55]. *Arcobacter* is also abundant in effluents from wastewater treatment plants [56]. Moreover, *Arcobacter* has shown resistance to several antibiotics [55] and it has been proposed to be involved in the exchange of resistance genes between gram-negative and gram-positive phyla [57] and to carry several virulence genes [58]. Notwithstanding, reports in Peru on the pathogenicity of this genus are scarce, e.g. we found only one published study that mentioned *Arcobacter* in stool from children with diarrhea [59].

The official Peruvian environmental quality standards for surface water evaluate only two microorganisms, *Escherichia coli* and *Vibrio choleare* [60]. Our results are aligned with previous work [61, 62] that corroborate that next-generation sequencing is capable to identify potential bacterial pathogens in the water samples. Thus, we believe that a future national water surveillance system shall include evidence from amplicon sequencing and metagenomics.

The high occurrence of bacterial chemotaxis pathway, a necessary function to move towards nutrients or away toxins, would be connected to bacterial growth and survival in aquatic environments [63]. A study in the Pinheiros river found similar high occurrence of bacterial chemotaxis and flagellar assembly functions and hypothesized that this could be influenced by the elevated concentration of nutrients, e.g. ammonia and phosphates [14]. In our study, one of the most predicted KO was the MCP protein (S7 Table). This protein is important for bacterial motility because it triggers the activation of flagella and has been also encountered in other highly polluted rivers such as the Yamuna, a major tributary of the Ganges river in India [64]. In addition, we found a high occurrence of predicted ABC transporters, ubiquitous proteins involved in several processes including nutrient uptake and chemotaxis [65]. According to the KEGG annotation these proteins also take part in quorum sensing functions. Quorum sensing has been profusely studied in free-living bacteria in laboratory conditions. However, it is now known that, in nature, bacteria can use quorum sensing mechanisms in fluid environments such as rivers, streams, intertidal and marine areas by forming biofilms [66]. We found the glutathione metabolism pathway was common in Santa Eulalia and Jicamarca-Santa Eulalia. Glutathione S-transferase is involved in biodegradation of xenobiotics, defense against chemical and oxidative stress, and antibiotic resistance [67]. Rivers have been shown as reservoirs of genes related to antibiotic resistance influenced by anthropogenic causes [64, 68]. Further research is necessary to obtain environmental metagenomes from the Rimac and to look for possible genes linked to resistance to antibiotics in the Lower Rimac or resistance to heavy metals in the Upper Rimac.

An untargeted GC-MS metabolomics coupled to multivariate statistical analysis showed differences in chemical composition between some localities from the Parac-Upper Rimac and Santa Eulalia-Parac with respect to the Lower Rimac. This result may be due to more population density in the Lower Rimac and to the distance from the Metropolitan area. Samples from the Lower Rimac clustered together in both, metabarcoding and metabolomics analyses (Fig 3, S3 Fig), suggesting similar factors such as pollution affecting bacterial and chemical compound diversity, especially, in the high populated Metropolitan area.

Additionally, compound identification was attempted for the 5 features of the discriminant panel. The GC-MS (APPI) analysis allowed generating elemental formulae for unknown compounds, which together with tandem MS capability contributed to their identification. Indeed, we found thiodicarb, 3-Indolebutyric acid and benzenemethanamine derivatives in the Parac-Upper Rimac and Santa Eulalia-Parac localities. The first compound is an agricultural insecticide against some species of Lepidoptera, Coleoptera and Hemiptera [69], the second one is a plant hormone, an ingredient in many commercial horticultural plant rooting products and a food-specific compound that was detected in human urine [70], and the third one is commonly used for the manufacture of plastic. Further sampling efforts should also consider the interaction between microbiota and chemical pollutants, product of anthropogenic activities [16].

Our study could be complemented with DNA metabarcoding analyses of other pathogenic protozoa [7] or invertebrates [71] which are also used as indicators of water quality. Additionally, it would be advisable to sample more localities between sites 5 to 8 to provide a better assessment of local diversity in the middle Rimac. Besides, we sampled in January 2020, rainy season in the Andes. Thus, it would be desirable collecting also during the dry season to compare temporal diversity patterns. Likewise, this research would be supported by other MS-based techniques like hyphenated and nonhyphenated MS to ensure the identification of family compounds in order to understand the complex interaction between microbial communities and environmental changes, natural or anthropogenic [16, 72, 73].

We showed that the integration of omics approaches with environmental sciences will be very beneficial to improve water quality assessments. We hope that the information from this work could be useful for further public health studies that could influence public policies on rural and sub-urban areas in Lima which depend on the Rimac river water. Finally, we believe that this initial work will promote more similar studies in our country and in the Latinamerican region.

## Supporting information

S1 Fig Venn diagram of most common KEGG orthologues (KO) shared among the Rimac sub-basins. (PDF)

S2 Fig Spearman correlation coefficient values. Bacterial ASVs frequencies were correlated with environmental parameters and significant identified chemical compounds. GC-MS: Gas chromatography—Mass spectomery. BD: Benzenemethanamine derivative. IBA: 3-indolebutyric acid.
(PDF)

S3 Fig Principal Component Analysis (PCA) diagram of metabolomics analysis of water samples from the Rimac sub-basins by GC-MS (APPI). (TIF)

S4 Fig Loading plot of PCA coefficients. The peaks represent metabolites described in S11 Table. Peak 22, Peak 23 (C7H19FN3OP), peak 29 (C7H14FOP), peak 30 (C9H19F2N5), peak 31, peak 43 (C12H21NS), peak 71 Benzenemethanamine derivative, C16H14BrN5), peak 87 (C11H14N3P), peak 92 (C13H7FN6S), peak 93 (Thiodicarb, C16H14BrN5), peak 104 (3-Indolebutyric acid, C12H13NO2), peak 112 (Benzenemethanamine derivative, C16H14BrN5), peak 119 (Benzenemethanamine derivative, C8H20F2N4OS4). These peaks were selected using a p-anova < 0.05 and total score > 95%. These peaks were selected using a p-anova < 0.05 and total score > 95%.
(TIF)

S1 Table Metadata associated with each sample. Summary of the original and filtered reads. Geographical location. Sample names. BioProject (SRA) accession codes.
(XLSX)

S2 Table Environmental and metabolomic parameters for each sample. We evaluated four parameters: pH, temperature, electric conductivity (EC) and nitrate concentration; and five peaks corresponding to the metabolites benzenemethanamine derivatives, thiodicarb, and 3-indolebutyricacid.
(XLSX)

S3 Table Frequency of ASVs in each sample. (XLSX)

S4 Table Taxonomic assignment for each ASV. (XLSX)

S5 Table Most common potential pathogens present in our samples. The biosafety information was obtained from the Risk Group database (American Biological Safety Association).
(XLSX)

S6 Table Description of KEGG orthologues (KO) present in the Rimac sub-basins. (XLSX)

S7 Table Metabolomic analysis by GC-MS (APPI). Data was normalized using the total ion current (TIC) and Log10-transformation.
(XLSX)

S8 Table Tentative identification of chemical compounds obtained by GC-MS for each in water samples from the Rimac sub-basins by GC-MS (APPI). Data was filtered using a score threshold > 95% and p-anova < 0.05.
(XLSX)
