# Supplementary figures and images for: From the Andes to the desert: 16S rRNA metabarcoding characterization of aquatic bacterial communities in the Rimac river, the main source of water for Lima, Peru
Source: PLoS One. 2021 Apr 22;16(4):e0250401. doi: 10.1371/journal.pone.0250401 (PMC8061919; doi:10.1371/journal.pone.0250401)

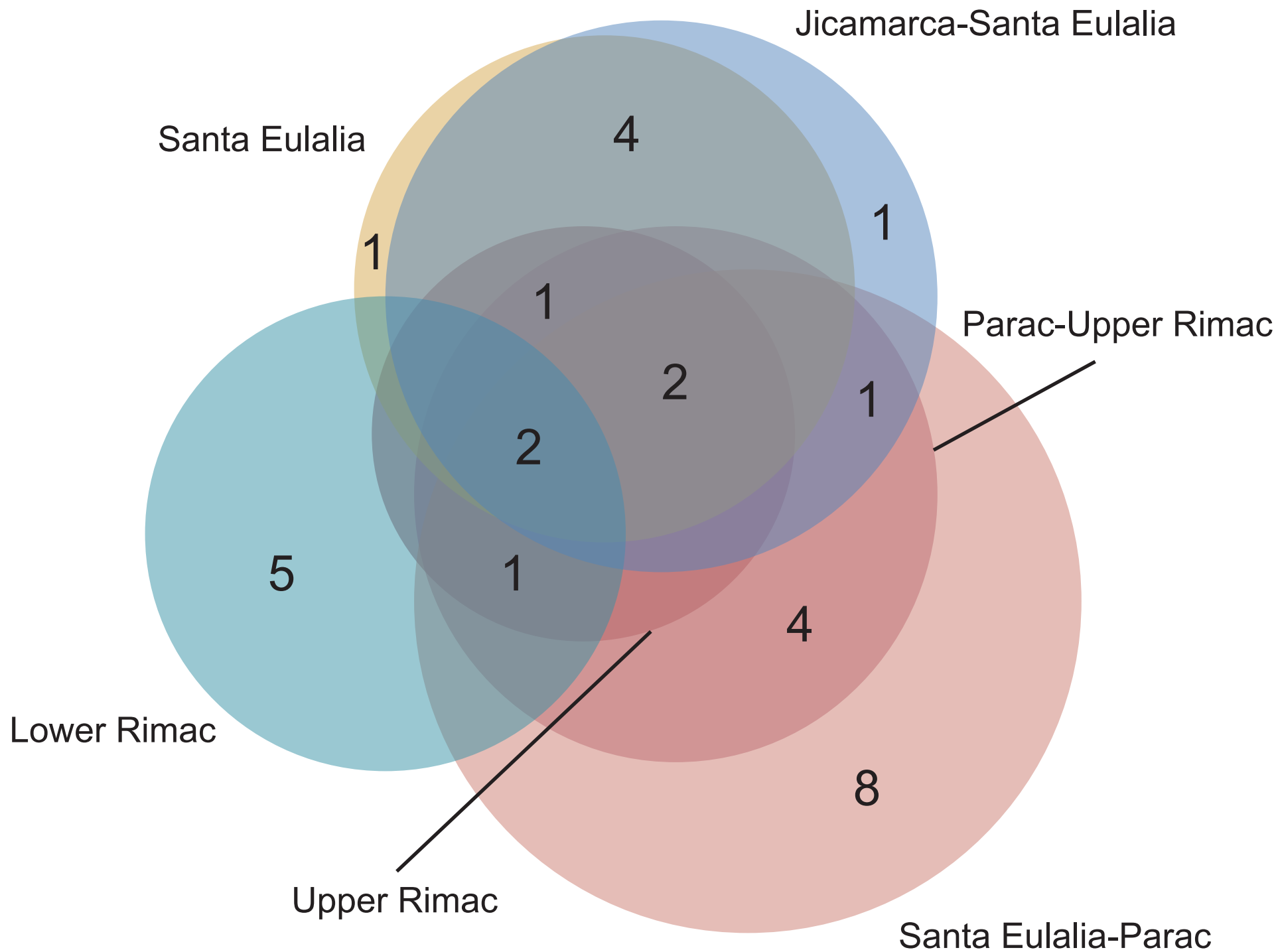

Supplement: S1 Fig — (PDF) [file pone.0250401.s001.pdf]

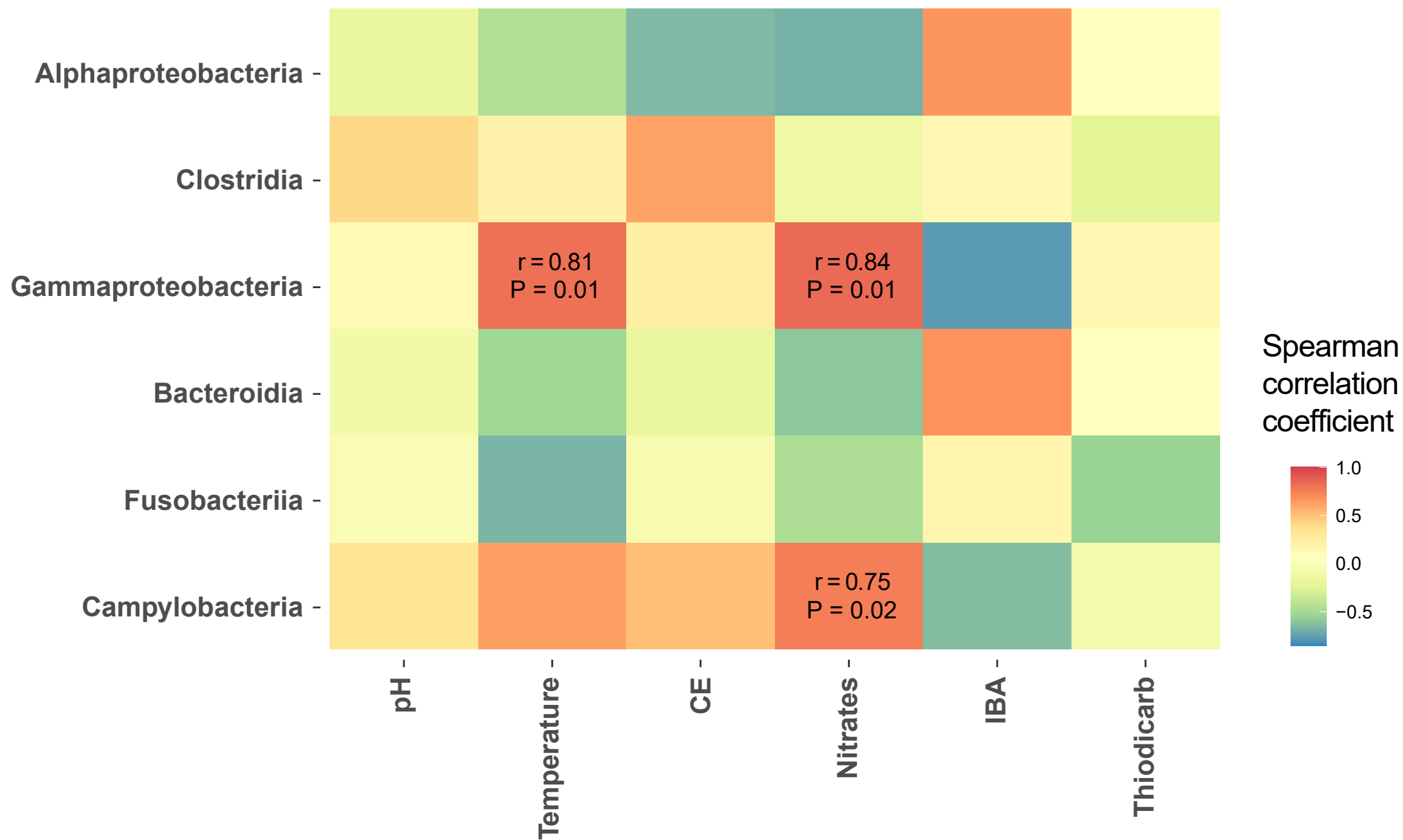

Supplement: S2 Fig — Bacterial ASVs frequencies were correlated with environmental parameters and significant identified chemical compounds. GC-MS: Gas chromatography—Mass spectomery. BD: Benzenemethanamine derivative. IBA: 3-indolebutyric acid. (PDF) [file pone.0250401.s002.pdf]

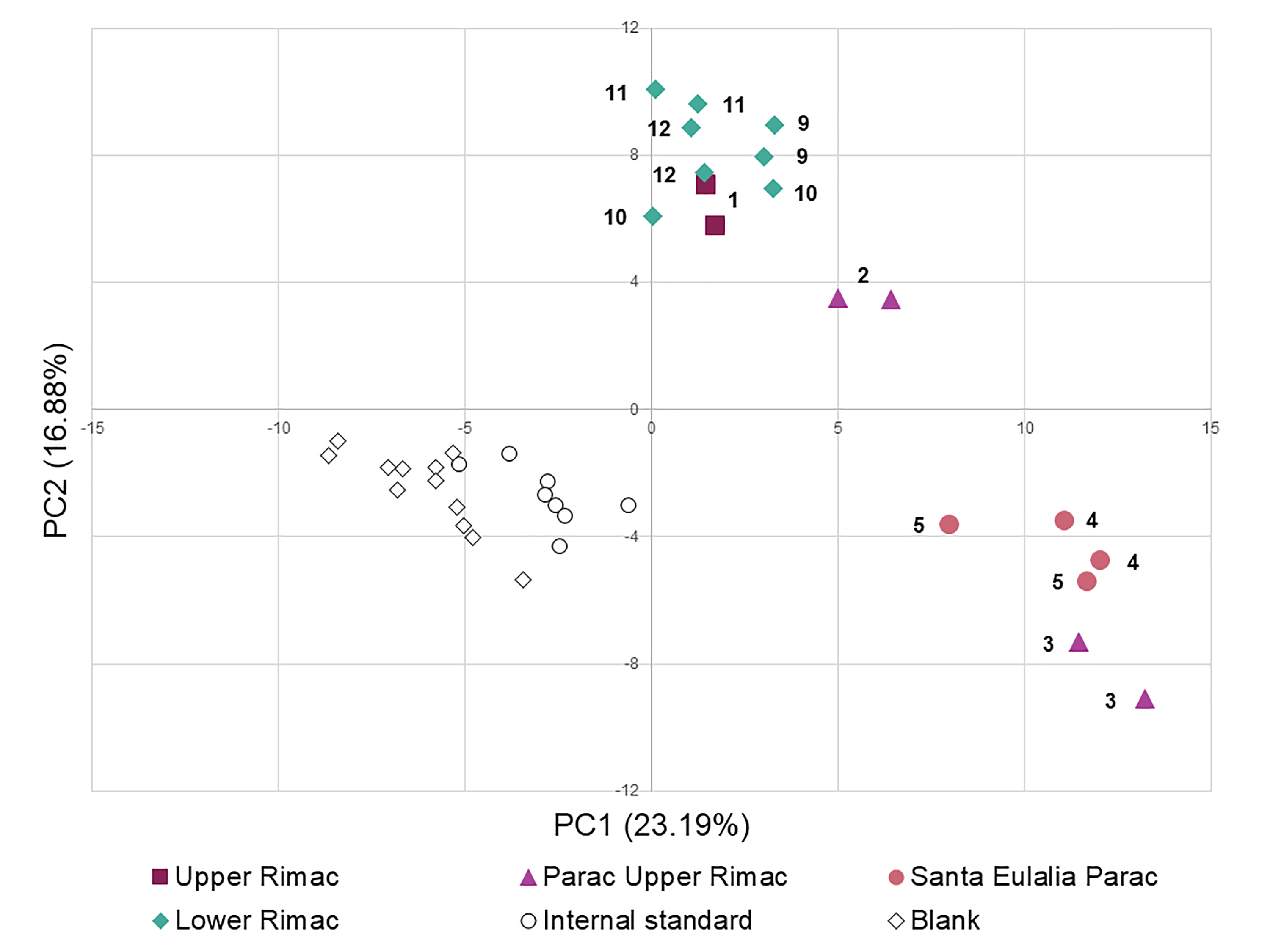

Supplement: S3 Fig — (TIF) [file pone.0250401.s003.tif]

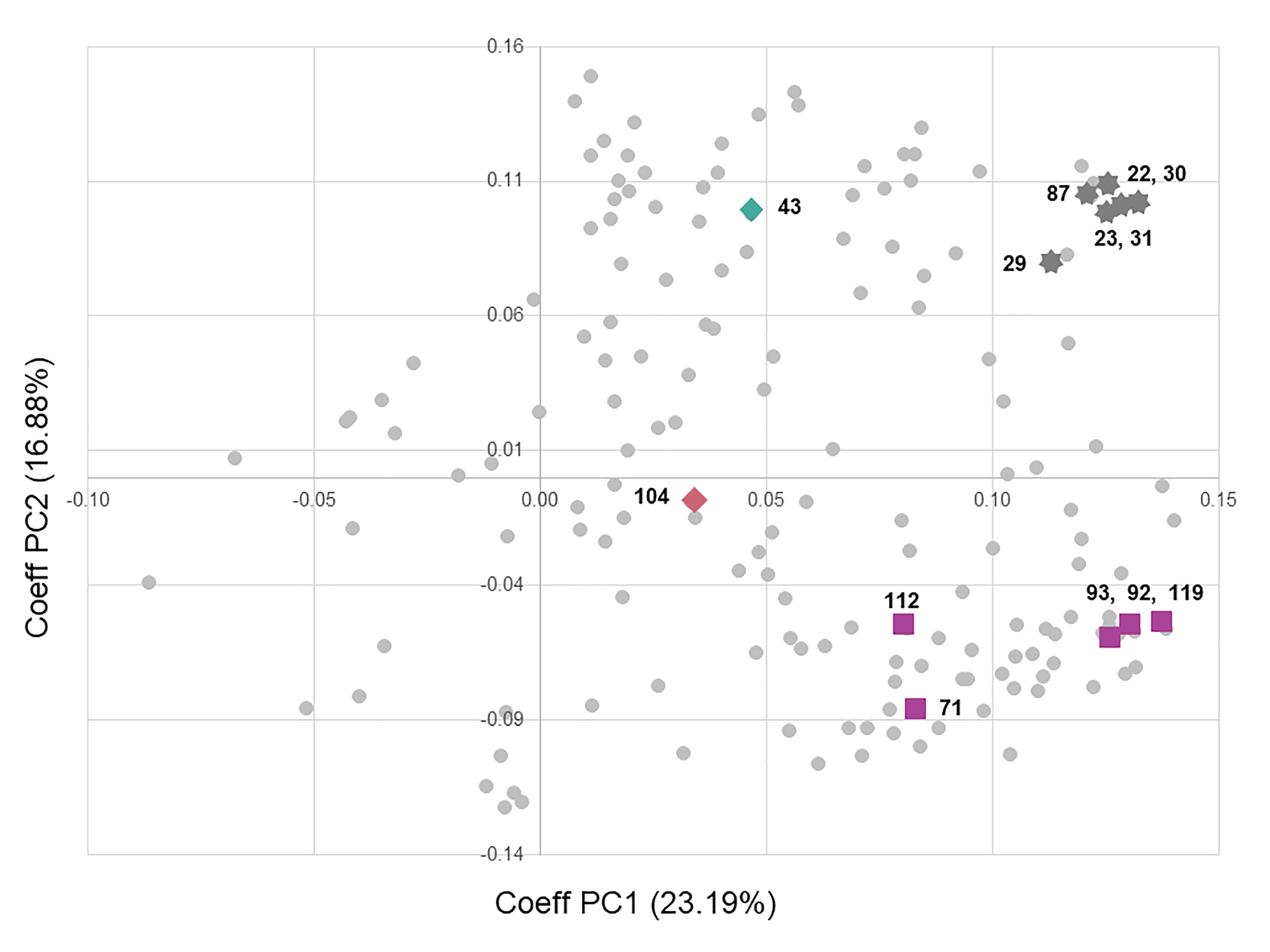

Supplement: S4 Fig — The peaks represent metabolites described in S11 Table. Peak 22, Peak 23 (C7H19FN3OP), peak 29 (C7H14FOP), peak 30 (C9H19F2N5), peak 31, peak 43 (C12H21NS), peak 71 Benzenemethanamine derivative, C16H14BrN5), peak 87 (C11H14N3P), peak 92 (C13H7FN6S), peak 93 (Thiodicarb, C16H14BrN5), peak 104 (3-Indolebutyric acid, C12H13NO2), peak 112 (Benzenemethanamine derivative, C16H14BrN5), peak 119 (Benzenemethanamine derivative, C8H20F2N4OS4). These peaks were selected using a p-anova < 0.05 and total score > 95%. These peaks were selected using a p-anova < 0.05 and total score > 95%. (TIF) [file pone.0250401.s004.tif]
